# Comparing Pain Intensity Using the Numeric Rating Scale and Defense and Veterans Pain Rating Scale in Patients Revisiting the Emergency Department

**DOI:** 10.7759/cureus.17501

**Published:** 2021-08-27

**Authors:** Sophia Sheikh, Jennifer Fishe, Ashley Norse, Morgan Henson, Divya James, Warren Sher, Michelle Lott, Colleen Kalynych, Phyllis Hendry

**Affiliations:** 1 Emergency Medicine, University of Florida College of Medicine – Jacksonville, Jacksonville, USA; 2 Pediatric Emergency Medicine, University of Florida College of Medicine – Jacksonville, Jacksonville, USA; 3 Emergency Medicine, University of Florida College of Medicine, Gainesville, USA

**Keywords:** dvprs, nrs, pain scores, emergency department, misuse, abuse, opioid epidemic

## Abstract

Objective

To determine the relationship between Numeric Rating Scale (NRS) and Defense and Veterans Pain Rating Scale (DVPRS) as pain intensity measures, we compared pain scores to sociodemographic and treatment data in patients revisiting the emergency department (ED).

Methods

After Institutional Review Board approval, 389 adults presenting within 30 days of an index visit were enrolled. Pain scores were classified as follows: 0-3 (mild), 4-7 (moderate), and 8-10 (high). Data were analyzed using descriptive analysis. Wilcoxon rank-sum test measured the association of pain score with gender. Pain scales were correlated using Spearman correlation coefficient. Pain scale association with opioid treatment was tested via ordinal logistic regression controlling for gender, home opioid use, and if ED revisit was for pain.

Results

Average patient age was 49. Most patients were African American (68.4%), male (51.2%), and returned for pain (67.0%). As continuous measures, both scales were positively correlated with each other (p<0.0001). Pain score severity categories were distributed differently between the two scales (p=0.0085), decreasing by 8% in patients reporting high pain severity when using DVPRS. For both scales, the proportion of patients (1) administered opioids (p=0.0009 and p≤0.0001, respectively) and (2) discharged with opioids (p=0.0103 and p=0.0417, respectively) increased with pain severity. Discharge NRS (p=0.0001) (OR=3.2, 1.780-5.988) and DVPRS pain score categories (p<0.0001) (OR=2.7, 95% CI=1.63-4.473) were associated with revisits for pain.

Conclusions

Our findings demonstrate a link between NRS and administration of opioid medications and suggest that DVPRS may better differentiate between moderate and high levels of pain in the ED setting.

## Introduction

Over 70% of patients presenting to the emergency department (ED) arrive with a pain-related chief complaint [1,2]. Assessing pain severity can be elusive due to its subjective multifaceted nature and basis in self-report. Several pain severity scales exist in attempts to quantify and characterize patients’ pain. One of the most widely used scales in medical practice is the unidimensional Numerical Rating Scale (NRS). Its use in assessment of pain intensity in acute to chronic pain has been reliable and validated in several populations and settings and correlates well with other pain scales employed in the assessment of pain intensity [2-7]. Features such as simplicity and rapidity of scoring and versatility in its mode of administration (including as a verbal assessment) explain why the NRS is a popular pain intensity scale in acute care settings like prehospital and the ED. However, unlike multidimensional pain scales, it does not factor the impact of pain on physical and emotional functioning. Patients using the NRS may exhibit a great degree of variability in interpreting and defining pain associated with each numerical value.

In contrast, the multidimensional Defense and Veterans Pain Rating Scale (DVPRS) combines functional assessment with pain intensity, assigning descriptive language, color coding, and visuals of faces to each number on its pain scale, drawing from the previously validated Faces Pain Scale Revised (FPS-R) [3,8,9] . The construct validity, internal consistency reliability, and test-retest reliability of the DVPRS have been established within military inpatient and outpatient populations [10,11]. However, its application within the civilian emergency department setting has not been described within the literature.

Implementation of NRS in the ED setting has been noted to increase the frequency of analgesic administration, with higher pain scores exhibiting increased likelihood of analgesia use [12]. While it is important to address pain, failure to fully characterize a patient’s pain or sole reliance on NRS to guide treatment has been associated with increased incidence of opioid adverse drug reactions, including oversedation [13]. While the NRS has been compared against other pain instruments, such as the Visual Analog Scale (VAS) and the Verbal Rating Scale (VRS), to our knowledge the correlation of the NRS with the pain intensity component of the DVPRS has not yet been studied within the literature [7,14]. To determine the relationship between DVPRS and NRS as measures of pain intensity, we compared pain scores to sociodemographics, opioid treatment data, and if ED return visit was for pain in patients revisiting the ED within 30 days of an index visit. Secondary outcomes included association of opioid use with DVPRS and NRS absolute and categorical ratings.

## Materials and methods

Study setting and enrollment

**Figure 1:**
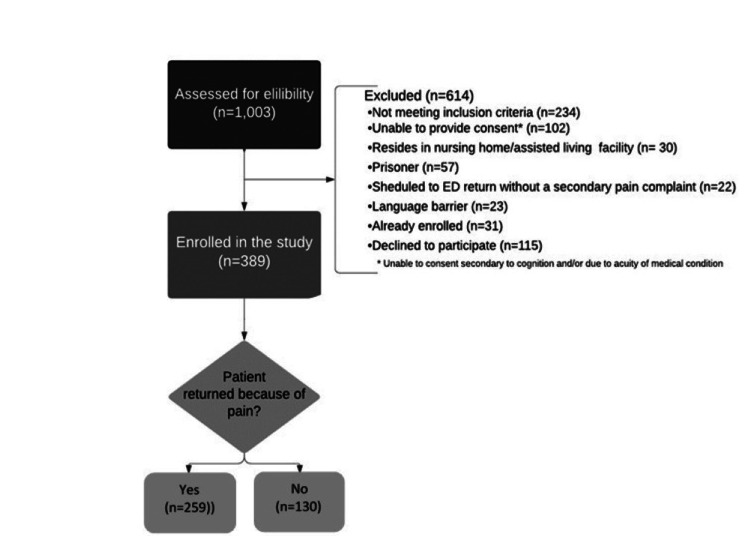
Patient Enrollment Schematic

Study enrollment occurred in the ED (annual patient volume over 70,000) of an urban safety-net hospital system. The average patient presenting to our ED is 50 years old, 51% are African American, 50% are male, and 25% are on Medicare. This study was a secondary analysis of data collected from the University of Florida Institutional Review Board-approved prospective observational study of 389 patients ≥18 years of age returning to the ED within 30 days of an initial (index) visit (Figure 1). Full study details have been published previously [15,16]. Briefly, all community-dwelling adult patients reporting to our ED and able to provide consent were eligible for inclusion. Patients not meeting these criteria and those instructed to return to the ED within a set time frame were excluded (e.g., scheduled wound checks or return for suture removal). Patients were enrolled using systematic time block sampling to mimic ED utilization rates by ED shift times based on historical data from our institution.

Variables collected

Sociodemographic variables, health literacy level, comorbidities, medication history, NRS and DVPRS pain scores, receipt of an opioid prescription, and reason for ED return within 30 days after discharge were collected from patient interviews and chart review. The Rapid Estimate of Adult Literacy in Medicine (REALM) was used to assess reading health literacy level (scores 0-18=third grade and below; 19-44=fourth to sixth grade; 45-60=seventh to eighth grade; and 61-66=high school) [17]. Pain scores were classified as follows: 0-3 (mild), 4-7 (moderate), and 8-10 (high).

Data analysis

Descriptive analysis was used to characterize demographics and clinical characteristics of the included study patients. Spearman correlation coefficient was used to describe the correlation of the pain scales with each other since they were not normally distributed. For the association of pain scale scores with male versus female gender, Wilcoxon rank sum tests were used. To test the association of the different pain scores with 1) ED opioid medication administration and 2) ED prescription of an opioid, we first attempted a linear regression. However, traditional transformations were unsuccessful in achieving a normal distribution for the pain scores. Therefore, we converted each pain score into an ordinal variable, with 0-3 equaling mild pain, 4-7 moderate pain, and 8-10 severe pain. We then performed an ordinal logistic regression controlling for gender, prior home opioid prescription, and if the ED revisit was for pain. All analysis was performed with SAS (Cary, NC), v9.4.

## Results

Study population characteristics

Tables 1, 2 display the study population characteristics. The mean patient age was 49. The mean health literacy level was seventh- to eighth-grade reading level. Of the 389 patients enrolled, 67% (259) of patients returned because of pain, 51% (129) for acute pain, and 18% (48) for chronic pain. In the group of patients returning to the ED for pain, 46% (119) reported use of opioid medications at home.

**Table 1:** Characteristics of Study Population (Categorical Variables) *Emergency Severity Index Triage Tool: acuity level 1: immediate; acuity level 2: emergent; acuity level 3: urgent; acuity level 4: semiurgent; and acuity level 5: nonurgent.

Variable	Category	Frequency
Gender	Female	190 (49)
	Male	199 (51)
Race	White	114 (29)
	Non-white	275 (71)
ED return visit reason	Pain	259 (67)
	Not pain related	130 (33)
ED acuity level*	1	2 (1)
	2	201 (52)
	3	173 (45)
	4	9 (2)
ED opioid treatment at revisit	No	241 (62)
	Yes	148 (38)
ED opioid prescription at revisit	No	342 (88)
	Yes	47 (12)
Disposition	Discharge	199 (51)
	Admit/observation	179 (46)
	Against Medical Advice/eloped	7 (2)
	Transferred	4 (1)

 

**Table 2:** Characteristics of Study Population (Continuous Variables) DVPRS: Defense and Veterans Pain Rating Scale, NRS: Numeric Rating Scale, REALM: Rapid Estimate of Adult Literacy in Medicine.

Variable	N	Mean	Standard Deviation	Median	First Quartile	Third Quartile
Age	389	49.4	15.4	51.8	37.8	60.1
Health literacy (REALM Score)	354	52.6	17.2	60	49	64
DVPRS pain score	373	6.6	3.2	8	5	9
NRS pain score	386	6.4	3.7	8	4	10
Total number of ED visits in 30 days	389	2.9	1.7	2	2	3

Comparison of pain scales at ED revisit: univariate analysis

As continuous measures, the NRS and DVPRS were positively correlated with each other (p<0.0001). Both the NRS and DVPRS were negatively correlated with age (p=0.0004 and p=0.0071, respectively). Pain scale severity was not associated with health literacy for either scale. Pain score severity categories were distributed differently between the two scales, particularly for the moderate and high pain severity groups (p=0.0085, Table 3).

**Table 3:** Comparison of Pain Scales by Pain Category Severity

	Low (0-3)	Moderate (4-7)	High (8-10)	Overall	p Value
Defense Veterans Pain Rating Scale	152 (39)	113 (29)	124 (32)	389 (100)	0.0085
Numeric Rating Scale	152 (39)	80 (21)	157 (40)	389 (100)	

Table 4 displays the relationship between categorical variables and pain severity categories for each pain scale at the time of ED revisit. Pain category severity was distributed differently by gender for the DVPRS but not for the NRS (p=0.02 and p=0.57, respectively). The proportion of patients who received opioid treatment in the ED increased as NRS and DVPRS pain category severity increased from low to high (p≤0.0001 and p=0.0009, respectively). DVPRS scores were distributed differently in patients discharged with prescription opioids from the ED (p=0.0417). The proportion of patients discharged with an ED opioid prescription increased as NRS pain category severity increased (p=0.0103). The proportion of patients administered opioid medications in the ED increased as the NRS pain category severity worsened at the time of ED discharge (p≤0.0001). The proportion of patients discharged with an opioid prescription also increased as the NRS pain category severity worsened at the time of ED discharge.

**Table 4:** Comparison of Categorical Variables by Pain Scale All tests were done using Chi-Squared unless indicated by *, which was analyzed using Wilcoxon Rank Sum. AMA: Against Medical Advice, DVPRS: Defense Veterans Pain Rating Scale, NRS: Numeric Rating Scale.

		DVPRS Category					NRS Category				
Variable (n, %)	Category	Low	Moderate	High	Total	p Value	Low	Moderate	High	Total	p Value
Gender*	Female	36 (20)	44 (24)	104 (57)	184 (49)	0.0200	50 (26)	32 (17)	108 (57)	190 (49)	0.5700
	Male	29 (15)	70 (37)	90 (48)	189 (51)		51 (26)	41 (21)	104 (53)	196 (51)	
Race	White	15 (14)	40 (38)	51 (48)	106 (28)	0.1500	25 (22)	28 (25)	61 (53)	114 (30)	0.1400
	Non-white	50 (19)	74 (28)	143 (54)	267 (72)		76 (28)	45 (16)	151 (56)	272 (70)	
ED opioid treatment	No	108 (45)	72 (30)	61 (25)	241 (62)	0.0009	122 (51)	60 (25)	59 (24)	241 (62)	<0.0001
	Yes	44 (30)	41 (28)	63 (42)	148 (38)		30 (20)	20 (14)	98 (66)	148 (38)	
ED opioid prescription	No	138 (40)	92 (27)	112 (33)	342 (88)	0.0417	143 (42)	66 (19)	133 (39)	342 (88)	0.0103
	Yes	14 (30)	21 (45)	12 (25)	47 (12)		9 (19)	14 (30)	24 (51)	47 (12)	
Disposition	Discharge	29 (15)	68 (34)	101 (51)	198 (53)	0.3890	40 (20)	39 (20)	118 (60)	197 (52)	0.8290
	Admit	35 (21)	44 (26)	89 (53)	168 (45)		58 (33)	32 (18)	88 (49)	178 (46)	
	AMA+/ eloped	1 (14)	2 (29)	4 (57)	7 (2)		1 (14)	1 (14)	5 (72)	7 (2)	

Comparison of pain scales at ED revisit: multivariable analysis

Increasing NRS pain category severity at triage predicted ED opioid administration (p<0.0001) (OR=2.7, 1.639-4.339). Increasing NRS pain category severity at ED triage was associated with prior home opioid prescriptions (p=0.03) (OR=1.7, 1.04-2.7) and if the ED return visit was for pain (p<0.001) (OR=4.8, 2.9-8.0). Increasing NRS pain category severity at the time of ED discharge was associated with whether the ED revisit was because of pain (p=0.0001) (OR=3.2, 1.780-5.988). Increasing DVPRS pain category severity was also significantly associated with ED revisits for pain in the multivariable model (p<0.0001) (OR=3.101, 95% CI=1.926-4.992). Neither scale predicted whether a patient received a prescription for an opioid medication at discharge.

## Discussion

In comparing the pain intensity component of the DVPRS to that of the NRS, we found several similarities between the two scales. Both scales had a similar relationship with age, and they both predicted ED returns for pain as pain category severity increased. Also, neither scale predicted receipt of an opioid prescription at the time of ED discharge in the multivariable model.

Although we found that the two scales were positively correlated with one another when they were used as continuous measures and when scores were grouped into severity categories (low, moderate, and high), we were better able to characterize the relationship between the two scales. First, the proportion of patients in the low pain group was the same for both pain scales. These patients reported a pain score between 0 and 3 regardless of which pain scale was used. Second, the proportion of patients in the moderate and high pain groups changed depending on the pain scale used. The proportion of patients reporting high pain scores decreased from 40% to 32% when using the DVPRS. There was also a corresponding increase of 21% to 29% in the proportion reporting moderate pain when using the DVPRS. This indicates that the DVPRS may discriminate between moderate and high levels of pain more effectively than the NRS.

We also found that increasing NRS pain category severity was associated with a three-fold higher likelihood of receiving opioid treatment in the ED. The NRS predicting ED administration of opioid medications in our patient sample demonstrates the power of pain scales to influence pain management, specifically opioid administration, in acute care settings like the ED. Others have also reported associations between unidimensional pain scales and increased analgesia administration and opioid adverse events [14,15]. Our results of increasing NRS pain category severity predicting ED opioid administration and DVPRS differentiating between moderate and high levels of pain coupled together suggest that patients may receive fewer opioid medications in the ED if the DVPRS (and not NRS) is used to measure pain intensity. These findings should be confirmed in a larger patient population.

There are several limitations to note in this study. This study recruited patients from only one site. Additionally, 71% of our participants self-identified as non-white and most had limited health literacy. While these factors may limit the potential to apply our results to other ED populations, our findings are important in that they are derived from a largely minority and socially vulnerable patient population who tend to be underrepresented in the pain literature.

## Conclusions

With this analysis, we demonstrated a clear link between NRS scores and administration of opioid medications. Additionally, our results suggest that the DVPRS may be better at differentiating between moderate and high levels of pain compared to the NRS in the ED setting. Further study should be conducted to confirm these findings and should be replicated in other ED populations, particularly in those who are underrepresented in the pain literature.
